# Influence of Sensory Properties in Moderating Eating Behaviors and Food Intake

**DOI:** 10.3389/fnut.2022.841444

**Published:** 2022-02-21

**Authors:** Ciarán G. Forde, Kees de Graaf

**Affiliations:** Sensory Science and Eating Behavior Group, Division of Human Nutrition and Health, Wageningen University and Research, Wageningen, Netherlands

**Keywords:** sensory, food choice, energy intake, texture, odor, taste

## Abstract

Sensory properties inform likes and dislikes, but also play an important functional role in guiding food choice and intake behavior. Odors direct food choice and stimulate sensory-specific appetites and taste helps to anticipate calorie and nutrient content of food. Food textures moderate eating rate and the energy consumed to satiation and post-ingestive metabolism. We summarize how sensory cues moderate intake, and highlight opportunities to apply sensory approaches to improve dietary behavior. Salt, sweet and savory taste influence liking, but also influence energy intake to fullness, with higher taste intensity and duration linked to lower intake. Psycho-physical studies show it is relatively easy to rank taste intensities at different concentrations but more challenging to discriminate fat contents, and fat discrimination declines further when combined with high-taste intensity. Fat has low impact on sensory intensity, but makes significant contributions to energy content. Combinations of high taste and fat-content can promote passive energy over-consumption, and adding fat also increases energy intake rate (kcals/min), reducing opportunities to orally meter consumption. Consumers adapt their oral processing behaviors to a foods texture, which can influence the rate and extent of energy intake. Understanding how texture influences eating behaviors and bolus formation, affords new opportunities to impact eating rate, energy intake and metabolic response to food. Food formulation has traditionally focused on composition and sensory appeal. Future research needs to consider the role of sensory properties in moderating consumer interaction with their food environment, and how they influence calorie selection, and shape our eating behaviors and intake.

## Introduction; Sensory Cues as *Functional* Food Properties

A foods sensory appeal is largely determined by the physical and chemical properties that are sensed before and during consumption, which informs initial acceptance and the degree to which a food will be consumed (1). High sensory appeal is proposed as the main reason for excessive energy intakes, yet dietary energy intake patterns are not dominated only by highly palatable foods, and most energy is consumed from staple foods and meals with diverse sensory properties. This suggests that palatability is only one dimension of food intake, and that the sensory properties of food play an important *functional* role in guiding intake behavior, beyond simply promoting “liking”. The senses of vision and olfaction are involved in the anticipation of food intake and direct sensory specific appetites and food choice. By contrast, hearing, taste, retro-nasal olfaction, texture perception and trigeminal stimulation are directly involved during consumption, and collectively inform the onset of satiation and the termination of energy intake (2). These sensory signals are integrated to form a dynamic perceptual impression of a food, which determines both our liking and intake behavior to the point of satiation (3). Sensory cues are operational before and during a meal, having a direct effect on satiation, with less of an impact on satiety (4).

This mini-review provides a summary of recent findings on the role of odor, taste and texture on calorie selection and energy intake. We highlight the differential role of smell and taste and texture perception in the initiation and termination of eating, and the sensory impact of fat in promoting higher energy intake rates. Food texture can also influence the oral phase of digestion and subsequent metabolic responses, and we highlight opportunities to apply an empirical understanding of the role of sensory cues to moderate food choice, intake and the eating behaviors associated with the healthiness of the food supply.

## Impact of Palatability, Odors, and Taste on Energy Selection and Intake

High palatability is a powerful incentive to eat, and the ingestion of “good tasting foods” has been linked to a multitude of positive emotions (5). A higher palatability increases the probability that a particular food will be chosen amongst a set of alternatives (6), and research has also shown that higher palatability leads to an increase in energy intake [i.e., (7)]. However, it is an oversimplification to assume that we only choose and consume foods based on palatability, and food intake decisions are influenced by a complex set of factors that go beyond palatability (1). Food choice and intake are influenced by a multitude of factors and we do not only consume our “most liked” foods (8). Highly palatable foods are often regarded as “treats”, and are consumed infrequently and so do not contribute disproportionately to higher energy intakes. For example, treat foods such as ice cream contribute a relatively small proportion (5%) to consumed calories (9). The majority of daily energy intakes comes from “savory-fatty” foods (10, 11) and staple foods (12) which often have relatively low hedonic valence, such as rice, potato or pasta, rather than indulgent “treat” products often characterized as junk foods or empty calories (13). Whereas, liking may predict choice, the effect of higher palatability on intake is not a linear relationship, with research showing the predictive relationship between liking and intake tends to diminish at higher levels of palatability. For example, in a field study with US soldiers, soldiers consumed 100% of served portions when the liking rating on the 9-point hedonic scale was either a “8” or a “9”, yet the relationship between liking and intake tended to plateau at ratings above score of 5–6 (6). Beyond their role in liking, research in from numerous controlled sensory-feeding behavior studies has highlighted the role of food sensory properties in calorie selection and intake.

Research has shown that food related ambient odors can increase sensory specific appetites and directly influence food choice (14, 15). For example, when customers at an experimental restaurant were asked to choose their meals from a menu, the proportion selecting a fruit dessert increased when choices were made in the presence of a non-attentively perceived “pear” odor, than when choices were made without an odor (16). The implication is that our response, particularly to unattended odors, are likely to play an important role in food choices. Similarly, when exposed to odors signaling a specific “taste” (i.e., sweet/savory) participants had a greater appetite for congruent sweet/savory food, compared to incongruent products, suggesting odors can induce “sensory specific appetites” that influence choice, independently of liking (17, 18). Although this effect has been reproduced many times, it seems limited to choice and despite early findings to the contrary (19, 20), odors do not seem to have a direct impact of energy intake in realistic consumption conditions (21, 22).

By contrast taste quality and intensity have been shown to moderate energy selection and intake. Consuming foods with higher *umami* intensity has been shown to reduce subsequent energy intake (23, 24), and foods with congruent savory-taste and protein content have been shown to enhance post-meal satiety (25, 26). Taste *quality* and *intensity* reflect the concentration of the taste substrate in the food environment, such that “sweeter” foods tend to contain more mono- and di-saccharides and salty foods contain more NaCl. However, there are also exceptions to this relationship, where fat (which is usually present as triacylglycerol), has a low sensory impact, but a large impact on the energy content of foods. Fat sensation affects mouthfeel, flavor release, and can directly impact the rate of the energy intake (27). In many cases the widespread use of low and no-calorie sweeteners now means there can be a strong taste signal in the absence of any sugar. Humans are largely blind to the primary macronutrients sources of energy we consume including starch, protein and triacylglycerol, which have little or no taste activity (13). As such, the sense of taste is influential in linking what is perceived during consumption with the positive post-ingestive consequences of food intake, and through repeated exposure, taste acquires a predictive capacity where we learn to imprint preferences and habitual eating habits via a reciprocal effect of flavor-consequence learning (28, 29).

Whether one taste quality is more satiating than another has been investigated based on anecdotal reports that “sweet” foods were wrongly believed to be less satiating than savory foods on a kcal for kcal basis, and may therefore promote increased energy intake. Early research on this topic showed no difference in the short term effect of sweet/non-sweet carbohydrates on subsequent satiety (30) and later findings support this showing that energy compensation is no different whether the energy taste quality was sweet or savory (31). This is further supported by research on taste and satiation, which showed that *ad libitum* intakes were equivalent for sweet and salty/savory tasting versions of the same meal (32).

Within a meal, the combined duration and intensity or “magnitude” of a taste may also affect the onset of satiation. Studies have investigated the impact of taste duration on *ad libitum* intakes, while maintaining a constant intake rate using a peristaltic pump. In one study, researchers measured individual concentration-pleasantness curves for salt in tomato soup, and exposed subjects to equally palatable low and high intensity salt during separate *ad libitum* test meals (33, 34). A longer oro-sensory exposure time and higher salt intensity combined to decreased food *ad libitum* intake by ~9%, though the oro-sensory exposure had a stronger impact than taste intensity. Vickers et al. showed that high yogurt sweetness was liked more than a low sweetness, but consumption showed the opposite effect, indicating that higher sweetness intensity led to earlier satiation (35). Lasschuijt et al. showed that higher taste intensity led to earlier meal termination, but as with previous findings, the effect of oro-sensory duration had a stronger impact than taste intensity (36).

## Sensory Contribution of “Fat”; Perceptually Benign but Energetically Potent

New techniques have been developed in recent years to profile dietary energy-intake behaviors based on the predominant taste properties of the foods consumed. This has produced new insights on consumer sensory-patterns of dietary intake based on the preferred taste quality of energy and nutrients consumed (37). This approach has been described as “*sensory-epidemiology”* and it enables comparison of daily energy intakes by clustering foods by their predominant taste quality, and then comparing the relative contribution of taste clusters to higher or lower energy consumed. Whereas, much attention has been directed to role of sweet foods and added sugar to high energy intakes, findings suggest that the excess energy intakes are mostly associated with greater intakes of “savory–fatty” tasting foods, which are consistently associated with increased energy intakes and higher rates of overweight (11). The implication is that foods high in “savory-fat” combinations make a significant contribution to daily energy intakes. Previous research has shown that our ability to discriminate between fat levels in food is reasonably linear at low taste intensity, but this ability dramatically decreases when fat is presented alongside higher intensity of sweet (38) or salty (39, 40) tastes. The implication is that when we are unable to detect increases in energy density due to higher fat, it becomes more difficult to adjust portion selected or later energy intakes in response to higher energy consumed from fat (41). It is therefore relatively easy to “hide” fats in foods without the fat being sensed, yet it makes a significant contribution to energy intakes. For example, *ad libitum* energy intake was ~2,100 kcal on a diet with 15–20% energy from fat compared to 2,600 kcal in a diet of equal palatability which derived 45–50% energy from fat (42).

Foods with a higher fat content can also lubricate and agglomerate more rapidly during consumption, which enhances bolus formation and increases eating rate and the extent of energy intake (43–45). As we summarize in the following section, this dual impact of increased eating rate and energy density can also promote excessive energy intakes.

## Food Texture, Eating Rate, Energy Intake, and Metabolism

Eating behaviors emerge in response to the texture challenges encountered during consumption, where consumers adapts their microstructural patterns of oral processing to prepare the initial structure for safe swallow (46). The effect of texture on satiation/food intake is mainly operational through eating rate, where harder, chunkier, more viscous textures result in lower eating rates (47). Previous research on liquid and semi-solid foods has shown that *ad libitum* intake of a liquid was ~30% higher than that of a semi-solid food (48). Difference in intake between liquid and semi-solids disappear when eating rates are set equal, with the help of peristaltic pump (49). There is wide natural in the eating rate of foods commonly encountered, with recent comparisons highlighting a range of between 10 and 120 g/min for solid foods, and rates of up to 400–600 g/min for liquids (50). Energy dense liquids pose a double-risk as they can be easily over-consumed, but also deliver poor satiety on a kcal for kcal basis (51).

Significant progress has been made in our understanding of how food texture influences oral processing (47, 52) and the specific influence of food textures on food intake (53). Food texture has been shown to drive eating rate (44, 54, 55) which in turn can influence *ad libitum* energy intake to satiation (56), and several studies have shown that faster eating is associated with the transition to overweight and obesity and poor cardio-metabolic health (55, 57–59). A meta-analysis of the food physical and sensory properties that affect intake concluded that people tend to consume less when solid-foods were harder, chunkier and more viscous (60). Evidence from numerous studies (53, 54, 61) now suggest that with a 20% reduction in eating rate, it is possible to reduce *ad libitum* energy intake by 1–14% without a loss in subsequent feelings of satisfaction (62). Food form and mode of consumption can also influence the rate and extent of intake, and solids have been shown to have a higher satiating efficiency than semi-solids and liquids, unless consumed slowly (as a soup) (63). Similarly, intakes were ~100 g lower each day when a semi-solid food was consumed with a spoon than a straw, highlighting that slower eating rate can support the onset of satiation for fewer calories (64). Food can influence eating rate but also oral processing and saliva-bolus uptake during the oral phase of digestion. Differences in food oral processing behavior have been shown to contribute to temporal changes in post-prandial glucose and insulin, and post-meal satiety responses (65–67). Slower eating rates result in greater bolus surface area, saliva uptakes and may have an incretin effect as early glucose release stimulates greater early insulin release (36, 65). Taken together, these findings indicate that food texture contributes much more simply “sensory appeal”, and can effect satiation and satiety by moderating eating rate, but can also exerts influence on the oral phase of digestion and the subsequent metabolic response to ingested nutrients. Further research is needed to understand how food texture based differences in eating rate can influence food intake control and support healthy metabolic responses to ingested nutrients.

## Future Opportunities for Sensory Nutrition

Public health guidelines recommend reductions in sugar, salt and fat but rarely consider the functional role of a foods sensory properties on choice and intake, or opportunities to incorporate an understanding of sensory cues in guiding reformulation or eating behavior changes. This review provides an overview of data that consistently shows how sensory cues have a reproducible influence on how we select, consume and feel satisfied from the foods in our diet. Further research is need to understand whether sensory properties can support sustained changes in eating behavior and promote healthier dietary patterns in the longer-term. Future product development and renovation requires significant reductions in several public sensitive nutrients (i.e., salt, sugar and fat) alongside enhanced nutrient density, to support better health and reduce the risk of diet related chronic disease. Understanding how consumers perceive and consume a food is central to the success of efforts to improve dietary behavior. We outline three potential opportunities for future applications of “sensory-nutrition” approaches to support improved eating behavior, dietary patterns and health.

*Using food odors to promote healthy food choice:* Research has highlighted the food odors stimulate sensory specific appetites, and are associated with recalled energy content and memory for foods. This may influence “foraging” behavior and is likely to support how we navigate the food environment when making choices on what to consume (68). Limited research to date has focused on the application of odor primes to encourage sensory-appetites and choice for healthier food products, and future research should aim to explore whether odor cues can be applied to stimulate consumer appeal and reinforce positive elements of healthy food choice and consumption.

*Application of low-calorie taste compounds to sustain the appeal reduced energy foods*: Extensive research has shown the impact of no- and low-calorie sweeteners (non-nutritive sweeteners, NNS) to support sugar reduction, and this has been particularly effective in removing sugar from soft-drinks. Numerous meta-analyses of experimental evidence highlight that applying non-nutritive sweeteners to reduce the sugar content of the diet can both lower dietary energy density and support clinically significant reductions in body weight [i.e., see (69)]. However, as demonstrated earlier, “savory-fatty” foods make a significant contribution to daily energy intakes (11), yet less research has been focused on how to sustain sensory appeal of savory foods with reduced energy density. In addition to the potential application of umami savory enhancers highlighted in the current review, recent findings suggest that *kokumi* may have the potential to enhance sensory appeal, increase calorie estimates, while supporting energy density reduction (70). These *kokumi* compounds are low calorie taste enhancers, often comprising tri-peptides and yeast extracts, that are known to enhance sensations of mouthfulness, continuity and complexity, often mimicking the sensory impact of fat. Preliminary findings demonstrate that addition of *kokumi* compounds can enhance sensory dimensions linked to calorie expectations, and promote higher estimated calories and expected fullness across a series of equi-caloric broths. Future research should further explore the potential of Kokumi compounds to support calorie reduction while maintaining product sensory appeal.

*Texture and Energy Density to reduce intake to satiation;* Evidence from several controlled feeding studies has demonstrated that energy density (71) and food texture (53) can independently and in combination influence the rate and extent of energy intake within meals. Findings from a recent RCT on ultra-processed foods highlights that higher energy intake rates (kcals/min) support sustained increases in *ad libitum* energy intake (72). These energy intake rates have been shown to vary widely within the food environment (27). Enhancing food texture in combination with energy density reductions combine to produce an 10–14% reduction in energy intakes, with no loss in meal palatability or post-meal satisfaction (53, 62). Further research is now needed to demonstrate the sustained effect of texture-energy density interventions on habitual energy intakes and subsequent energy balance.

## Conclusions

Knowing that the sensory properties of food influence choice and intake behavior is important, but this knowledge will have little impact if we do not apply sensory cues to encourage the consumption of healthier diets. As illustrated above, a number of proof of principle studies have clearly shown that it is possible to change sensory cues in the food environment in such a way that people consume less calories while maintaining the palatability of diets. These approaches require further research to understand the longitudinal impact of sensory properties on energy intake in the food environment and across a wider population of consumers. Controlled “sensory-nutrition” intervention studies are required to further understand how effective these longer term approaches are in producing sensory optimized foods that help to moderate the flow of energy though our diets.

## Author Contributions

CF and KG conceived and wrote the manuscript. Both authors revised and approved the submitted version.

## Funding

The writing of this article was partially funded by the Dutch TKI-Agri-food Project Restructure; 2020-221.

## Conflict of Interest

CF is currently a member of the Global Scientific Advisory Committee of the Kerry Health and Nutrition Institute. KG is a member of the Scientific Advisory Board of Sensus BV (NL) and a member of the Scientific Advisory Board of the Cosun Nutrition Center (NL).

## Publisher's Note

All claims expressed in this article are solely those of the authors and do not necessarily represent those of their affiliated organizations, or those of the publisher, the editors and the reviewers. Any product that may be evaluated in this article, or claim that may be made by its manufacturer, is not guaranteed or endorsed by the publisher.
